# Supplementary material for: MetaRibo-Seq measures translation in microbiomes
Source: Nat Commun. 2020 Jun 29;11:3268. doi: 10.1038/s41467-020-17081-z (PMC7324362; doi:10.1038/s41467-020-17081-z)
Supplement: Supplementary file 10 — Supplementary Data 7 [file 41467_2020_17081_MOESM10_ESM.zip › File2/Confidence_High_Taxonomy/212295_out.krona.html]

Javascript must be enabled to view this page.

members
magnitude
magnitudeUnassigned
count
unassigned
taxon
rank

212295\_out

2

2
2
superkingdom

phylum
1239
2

186801
class
2

186802
order
2

2
family
186803

189330
genus
2

species
1262873
2

SRS077294\_contig\_number\_contig-100\_42359.42359SRS148721\_contig\_number\_contig-100\_38153.142359
